# Self-Monitoring Practices and Use of Self-Monitoring Technologies by People with Rheumatic and Musculoskeletal Diseases: An International Survey Study

**DOI:** 10.3390/healthcare12191960

**Published:** 2024-10-01

**Authors:** Pedro Matias, Sílvia Rêgo, Francisco Nunes, Ricardo Araújo, Nadja Kartschmit, Tanita-Christina Wilhelmer, Tanja Stamm, Paul Studenic

**Affiliations:** 1 Fraunhofer Portugal AICOS, Rua Alfredo Allen 455/461, 4200-135 Porto, Portugal; pedro.matias@fraunhofer.pt (P.M.); silvia.rego@fraunhofer.pt (S.R.); francisco.nunes@fraunhofer.pt (F.N.); ricardo.araujo@fraunhofer.pt (R.A.); 2 Center for Medical Data Science, Institute of Outcomes Research, Medical University of Vienna, 1090 Vienna, Austria; nadja.kartschmit@meduniwien.ac.at (N.K.); tanja.stamm@meduniwien.ac.at (T.S.); 3 Österreichische Rheumaliga, 5761 Maria Alm, Austria; tanita.christina@gmail.com; 4 Division of Rheumatology, Department of Internal Medicine III, Medical University of Vienna, 1090 Vienna, Austria; 5 Division of Rheumatology, Department of Medicine (Solna), Karolinska Institutet, 171 77 Stockholm, Sweden

**Keywords:** RMDs, patient-reported outcomes, self-monitoring, health-related quality of life, quality of care, digital health applications

## Abstract

Background/Objectives: Digital health applications (DHAs) promise to improve disease self-management, but adherence remains suboptimal. We aimed to explore self-monitoring practices of rheumatic and musculoskeletal diseases (RMD) patients. A web-survey was conducted over 7 months including RMD patients to study their self-monitoring practices and the potential of DHAs. Methods: Health, sociodemographic, and technology adherence indicators were retrieved for comparison. Regression analyses and unsupervised profiling were performed to investigate multiple patient profiles. Results: From 228 responses gathered, most reported willingness to use DHAs to monitor their condition (78% agreement), although the majority rarely/never tracked symptoms (64%), often due to stable condition or no perceived value (62%). Of those tracking regularly, 52% used non-digital means. Participants with regular self-monitoring practices were more open to use a self-monitoring app (OR = 0.8 [0.6, 0.9]; *p* = 0.008) and be embedded in multidisciplinary care (OR = 1.4 [1.1, 1.6]; *p* < 0.001), but showed worse health status (g = 0.4; *p* = 0.006). Cluster analyses revealed three distinct groups of reasons for not tracking regularly ($\chi^{2}$ = 174.4; *p* < 0.001), two characterised by perceived low disease activity. Conclusions: Effective use of DHAs remains limited and non-digital means prevail in symptom monitoring. Findings suggest that better patient engagement strategies and passive monitoring should be adopted in early development stages of DHAs for better long-term disease self-care.

## 1. Introduction

Rheumatic and musculoskeletal diseases (RMDs) are a major cause of disability and encompass a broad range of conditions that affect joints, muscles, bones, and other organ systems. Symptoms are characterised by pain, swelling and stiffness of joints, as well as damage to bones and joints, often leading to progressively increased disability [1,2]. RMDs affect roughly 16% of people in Europe [3], being projected to rise due to population ageing [2,4,5]. The care of RMD patients usually includes regular visits to the clinician [6], spanning from every 3 to 4 months for inflammatory (e.g., rheumatoid arthritis), or 6 to 10 months for non-inflammatory conditions (e.g., osteoarthritis) [7]. Within these clinical visits, disease activity is assessed with physical and laboratory examinations, and clinical history analysis, ideally supported by patient-reported outcome measures (PROMs) [8].

Optimal patient care may, however, be compromised due to the ever-growing healthcare demand and professional shortages [1]. In Canada, the USA, and the UK, shortages are already impacting patients with RMDs [2], and the American College of Rheumatology estimates that, by 2030, demand for rheumatology services will exceed their capacity by 102% [2]. Patients might expect to experience progressively worse health, quality of life (QoL), and debilitating pain due to less frequent in-person outpatient appointments [2]. To minimise impact, the European Alliance of Associations for Rheumatology (EULAR) recommends using telehealth-based follow-ups and self-management interventions (e.g., symptom monitoring) to minimise impact on patients and increase efficiency of in-person visits [2]. Engaging in further self-monitoring will require patients to effectively track mobility, pain, fatigue, and disease activity using PROMs or passive sensing [9].

Previous studies have shown that the percentage of patients with RMDs performing self-monitoring is still quite low [9]. A survey study carried out by Kernder et al. [10], found that RMD patients show low usage of digital means for self-monitoring their condition. To the best of our knowledge, there is little information disclosed in the literature about the technology use and actual self-monitoring practices of patients living with RMDs. Previously, it was reported that patients would be open to using digital health applications (DHAs) [11], as these had potential to support the self-management of rheumatic diseases [12,13], facilitating the systematic collection and visualisation of disease-related data [12,13]. In addition, these apps can analyse and display data [2,14,15,16], so patients can visualise trends that help in reflections of self-awareness of their own behaviours. This may ultimately help act towards better treatment adherence and symptom management, which is a key element in clinical self-care [17,18].

The adherence to self-monitoring apps remains a challenge [18,19], with some studies reporting that the main barriers are related to technical factors, such as the system login or the PROM reminder alerts [18]. Usability of interfaces plays a particularly important role in the interaction with DHAs by individuals with RMDs, which is partly compromised due to pain and disability in body extremities (e.g., hands) [20]. However, patient perspectives have been disregarded in the conduction of studies with DHAs for RMDs, [6,11,21] as patients have rarely been involved in the early development stages of such apps [22]. This knowledge enables anticipating needs and barriers to be addressed during such phases to facilitate long-term adherence [19], ensure usability [11,20], and successfully integrate into clinical care [11], to benefit patients. For this reason, the EULAR and the Young Rheumatology Working Group of the German Society for Rheumatology (Rheumadocs) recommend integrating patients’ insights in the design of DHAs [6,11].

The literature lacks comprehensive research on specific self-monitoring practices and tools used by patients with RMDs. Adherence and usability to DHA-based solutions remains a challenge, which demands further research. Thus, in this study, we aimed to comprehensively, (1) obtain the perspectives and practices of people living with RMDs regarding self-monitoring, (2) associate self-monitoring with disease activity levels, health-related QoL, and technology adherence, and (3) assess both the potential of DHAs and their most important features to support patients in their disease self-monitoring and self-care.

## 2. Materials and Methods

**Figure 1:**
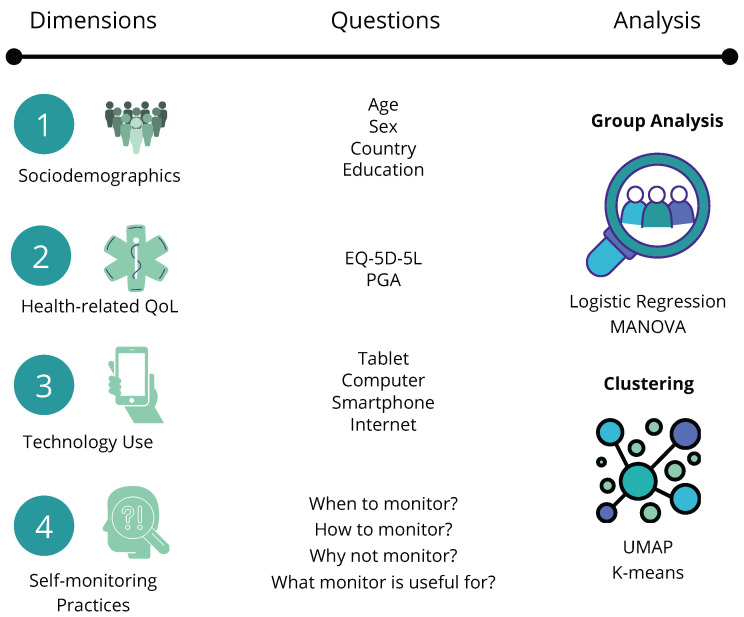
Illustration of the study design. Dimensions collected, questions asked, and analysis performed are described.

This web-survey was designed and carried out based on established standards to disseminate the results in a reproducible, validated, and transparent fashion. Figure 1 helps illustrate the data collected and the methodology adopted for analysis.

### 2.1. Study Design

The study reports our analytical findings from an online survey including patients with RMDs. The survey targeted a European population (with emphasis on people from Austria, Germany, Ireland, and Portugal), whose data were collected between September 2022 and March 2023. It comprised 32 questions in total, with 4.3±2.2 items/page (M ± STD). The questionnaire collected data on the following: (1) sociodemographics, (2) health-related quality of life [23], and patient global assessment (PGA) [24], (3) general technology usage, and (4) disease self-monitoring practices.

### 2.2. Survey Question Design

Questions were always displayed in the same sequential order (see Appendix A). They were formulated in English by the study team based on relevant domain knowledge and iteratively discussed with patient research partners (PRP). After translating the questions into German and Portuguese, a group of researchers and patients was invited to test the questionnaire, which resulted in cultural language adaptations to enhance question clarity. The data collection of personal information was minimised for privacy purposes. Participants could review their answers before submitting the survey.

### 2.3. Patient and Public Involvement

This study was part of the publicly funded COTIDIANA project [25] that integrated PRPs from the beginning of starting work. The collaboration with PRPs was carried out in different stages, consisting of pre-discussing survey items, providing input to the survey questionnaire, adjustments to translated versions, discussing the results of the survey, and help in shaping and revising the manuscript.

### 2.4. Ethical Considerations and Recruitment

The present survey was approved by the Ethics Research Committee of NOVA Medical School (NMS|FCM-UNL, No. 149/2021/CEFCM) in Lisbon, Portugal. Recruitment was conducted through the Austrian Rheumaliga (ÖRL) and the Portuguese League Against Rheumatic Diseases (LPDR) communication channels. The survey was also shared in RMDs-associated Facebook groups, and by some involved researchers, on Twitter (currently X). In a second phase, individuals were invited to answer the survey upon advertisement through leaflets or poster at the outpatient clinic of the Division of Rheumatology at the Vienna General Hospital (AKH). The participation in the survey was voluntary and informed consent was digitally retrieved before starting the survey. Access to the survey was made through a shared link (no login required). No filter was used to prevent responses from the same IP address or browser; however, upon a response matching examination, it was determined that there were no entries that could be identified as originating from the same participant.

This survey was released through an online survey hosting platform (www.soscisurvey.de, accessed on 29 December 2023), which is fully compliant with the General Data Protection Regulation (GDPR) and the Federal Data Protection Act (FDPA).

### 2.5. Data Collection

Characterisation data included (1) age, gender, educational background, geographical location, and disease-related background (e.g., diagnosed conditions, follow-up period, time of symptom onset). General health status was assessed using the (2) EQ-5D-5L questionnaire [23], a validated instrument that asks respondents to rate their level of difficulty in performing activities from specific dimensions (e.g., mobility, usual activities, pain, anxiety), using a scale of 1 to 5. The higher the score of the domains, the poorer the outcome. The EQ-5D-5L index value summarises the participant’s health-related quality of life (QoL), usually ranging from 0 to 1, with 1 representing perfect health and 0 representing death (negative values may indicate very bad quality of life conditions, worse than death). Other questions to assess health status included physical activity levels, patient global assessment referring to the past week (0–100 PGA, higher scores indicate worse perception) [24], subjective health status (0–100 VAS, higher scores indicate better perception), and whether the disease is under control or not (yes vs. no). Technology usage was inferred from (3) the usage levels of multiple devices (smartphone, tablet, computer) and Internet. Finally, self-monitoring practices were enquired in terms of (4) how participants keep track of their health condition. These questions addressed (a) how they engage in self-monitoring, (b) when they track, (c) how often they measure, (d) why they do not engage in further monitoring, (e) which devices they use, (f) what the advantages of tracking symptoms are, and (g) the potential benefits of digital technologies for managing rheumatic diseases.

### 2.6. Statistical Analysis

Statistical analyses were conducted by identifying groups of interest and exploring associations between self-practices, health status, and technology use. In this work, as the focus was on investigating disease self-monitoring practices, question items asking respondents (a) if their disease was under control (yes vs. no) or (b) the frequency of symptom tracking (at least once a month vs. every 6 months or less) were evidenced for building associations with health status, technology adherence, and sociodemographic responses.

Logistic regression (or chi-square and Multivariate ANalysis of VAriance, MANOVA) and Mann–Whitney U statistical analyses assessed the discernability of categorical (nominal or ordinal) and continuous variables, respectively. Logistic regression evaluated single/multiple choice items from health-related, tech-related, and self-tracking sub-fields, whereas the Mann–Whitney test analysed continuous health assessment scales. Pearson’s correlation computed pairwise correlations seeking for consistency checks (e.g., between health assessment scales) and potential bias sources (e.g., in demographic items). The results from group analyses were adjusted for age, sex, and education level. Significance values were adjusted for multiple comparisons using the Benjamini–Hochberg False Discovery Rate (FDR) correction. Unsupervised analyses, via uniform manifold approximation and projection (UMAP) feature projection [26], were also performed to identify unknown groups with distinct answer patterns in self-monitoring items. A 2D non-linear projection was applied to the N-dimensional response profiles from self-monitoring items, after which a k-means clustering technique [27] was used to build sub-group cluster profiles.

Group splitting was conducted so that the resulting sample distribution was as balanced as possible. The significance values (at 5%) were reported alongside the magnitude of the effects (odds ratio OR, and Hedges g) for every item analysis. The OR score assumes only positive values, with values greater than one indicating a positive effect (negative otherwise), where the further it is from one the larger the effects. The Hedges g score extends from negative to positive infinity (the sign points towards the effect direction), where higher absolute values indicate larger effects. Data incompleteness was handled by rejecting from the analysis items with more than 20% of non-intentional missing values (e.g., errors). Analyses were conducted in Python v3.8 (pingouin v0.5.3 [28], confounds v0.1.3 [29], pandas v1.5.3 [30] packages). Visualisations were generated using the plotly v5.15.0 package [31].

## 3. Results

The population surveyed in this study consisted of 228 RMD participants. The completion rate (participants who completed the survey after starting it) was 97.44%, showing a very reduced number of dropout respondents. Participants took 8.92 ± 2.58 min to complete the survey. Most participants were female subjects (81.1%). Regarding age, 33.3% were 18 to 40 years old, 50.4% between 40 and 60, and 16.2% above 60. Geographically, the surveyed population was primarily from Austria (50.0%), followed by Portugal (35.5%), with smaller representations from Germany (5.3%), Ireland (4.8%), and other countries (4.4%). A great part of the respondents had inflammatory arthropathies (RA, 35.5%; SpA, 18.0%; PsA, 14.0%), and access to different healthcare providers during the last year. The majority also considered having their condition under control (73%). In Table 1, the characterisation of this survey’s population is represented by sociodemographic, clinical, and quality of life items. Refer to Appendix A.1 for details on the health-related QoL assessed from participants.

### 3.1. Usage of Technology Devices

Participants had high levels of technology engagement, with most respondents utilising smartphones (97%) and computers (75%) at least a few times per week. About 62% reported rather low usage of tablet devices (52% did not use and 10% only used them monthly). The Internet was accessed regularly by nearly all the respondents (97%). Notably, nearly all participants incorporated smartphones and computers into their daily routines, though this does not inherently imply using these devices for self-monitoring their health condition.

### 3.2. Self-Monitoring Practices of Patients with RMDs

**Figure 2:**
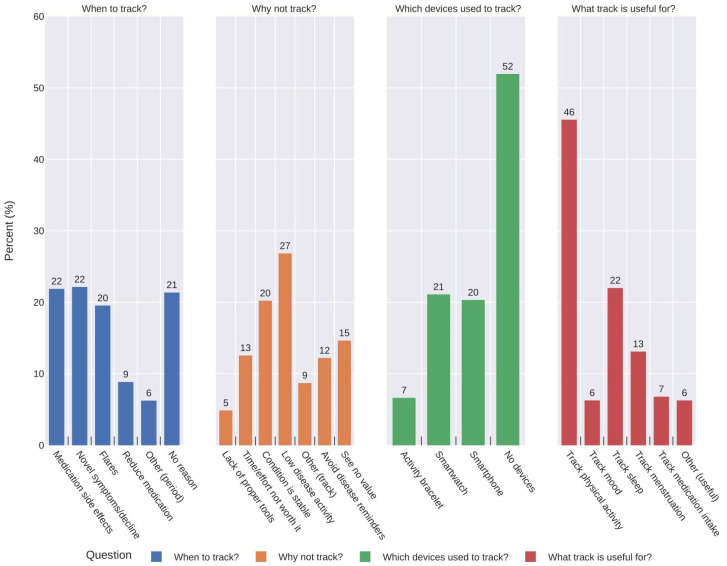
Response profiles on disease self-monitoring practices of respondents. The four bar charts represent (1) situations in which participants record symptoms, (2) why they do not track, (3) which devices they use to track, and (4) what tracking is useful for (from left to right).

Of 228 respondents, nearly 52% made use of non-digital approaches, monitoring their condition through mental notes (27%), paper notes (17%), or a diary (8%). Only 23% of participants used digital means or devices to self-monitor their condition, making use of mobile apps (15%), excel spreadsheets (6%), and web apps (2%). The remaining participants reported other approaches (5%) or simply did not track their symptoms (20%). By analysing the devices used to self-monitor their condition (n = 228), the majority did not use any devices (52%), while, for others, smartphones and smartwatches were top-listed. Those using devices described their usage mostly for tracking physical activity (46%) or sleep (22%), as displayed by Figure 2.

When asked how often they recorded condition-related symptoms, 36% answered daily or a few times per week (11%), weekly (12%), or monthly (13%), while the remaining (64%) rarely (once in six months) or never. Those who did track once a week or less (n = 203) were asked why. Most of the respondents (47%) mentioned their condition was stable (20%) or had low disease activity (27%), so did not need to track, while the remainder had not perceived value from tracking (15%), considered it too much effort (13%), or did not want to be reminded of their disease (12%). Typically, changes to treatment or condition status may often trigger self-monitoring practices (see detailed in Figure 2).

**Figure 3:**
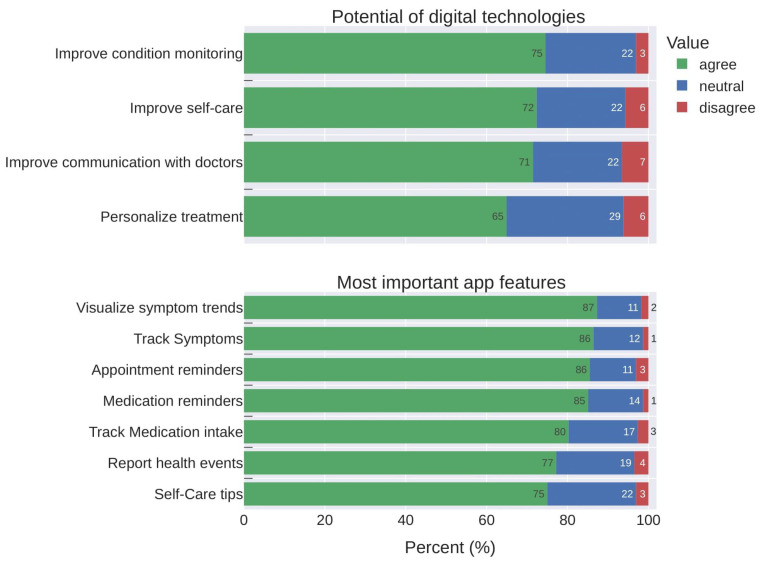
Levels of agreement of respondents when asked about (**top**) the potential benefits of digital health technologies and (**bottom**) which features a self-monitoring app should have.

Concerning the potential benefit of digital technologies, the majority agreed that it improves condition self-monitoring, self-care, and patient–doctor communication. In terms of the importance of certain app features, symptom tracking, trend visualisation, and reminders of appointments/medications were the most accepted (see Figure 3).

When asked about whom tracking information should be shared with (n = 224), the majority of participants (71%) included health professionals, such as rheumatologists (32%), family doctors (21%), physiotherapists (11%), and psychiatrists (7%). The remaining (29%) believed app information should be shared with family members and friends (16%), other app users (4%), and others (5%). Only a minority (3%) did not want to share data with anyone.

### 3.3. Group Analysis on Differentiating Factors of Self-Monitoring Practices

Participants who regularly record their symptoms (at least monthly) were more positive about the potential benefits of mobile apps for condition monitoring (OR: 0.7 [0.5, 1.0], *p* = 0.050), patient–physician communication (OR: 0.7 [0.5, 1.0], *p* = 0.049), and symptom tracking (OR: 0.6 [0.4, 0.9], *p* = 0.007). They used more devices (OR: 3.4 [1.5, 8.0], *p* = 0.004), also monitoring symptoms through more diverse means (OR: 2.4 [1.3, 4.3], *p* = 0.003) and in more specific situations (OR: 3.1 [2.2, 4.4], *p* < 0.001). Reporting in more situations was strongly correlated with respondents holding a higher educational degree (r = 0.87, *p* < 0.001). In addition, participants who did not self-monitor regularly were more reluctant to use an app for self-care (OR: 0.8 [0.6, 0.9], *p* = 0.008). The willingness to share symptom information was independent of the regularity of using an app for self-care (see Table 2).

Participants who considered their RMD under control (n = 165, stable condition) reported less regular symptom monitoring practices than those with more condition-related issues (OR: 0.8 [0.7, 1.0], *p* = 0.045), as depicted in Table 2. Interestingly, both groups used similar devices for either general use or symptom recording (*p* > 0.05). Among respondents who did not record symptoms regularly, those with a self-perceived stable condition often mentioned that having low disease activity was the main reason for not engaging in self-monitoring (OR: 0.1 [0.0, 0.2], *p* < 0.001), while participants perceiving their RMD was not under control mentioned the lack of access to proper tools (OR: 3.1 [1.0, 9.6], *p* = 0.047). In terms of physical activity (n = 228), more active individuals were willing to share their app-recorded symptoms with a wider number of specialists/family members (OR: 1.3 [1.1, 1.6], *p* = 0.001). However, distinct activity levels were not found to affect either the symptom monitoring frequency, the devices used for monitoring, or the levels of willingness for using a condition-supporting app (*p* > 0.05). Participants with a stable condition also showed higher physical activity levels (OR: 0.6 [0.4, 1.0], *p* = 0.026), fewer issues in every EQ-5D-5L dimension, and better levels of health status from EQ-VAS (g = −1.2, *p* < 0.001), PGA (g = 1.1, *p* < 0.001), and EQ-Index (g = −0.7, *p* < 0.001) scores (see Table 3).

Participants with regular monitoring habits displayed significantly worse EQ-VAS (g = 0.4, *p* = 0.015), PGA (g = −0.3, *p* = 0.028), and EQ-5D-5L scores (most items) than those with irregular symptom monitoring (Table 3). The EQ-Index also displayed lower (worse) levels in respondents tracking their symptoms regularly (g = 0.4, *p* = 0.015). Participants from the latter group were also followed by a wider variety of medical specialists (OR: 1.4 [1.1, 1.6], *p* < 0.001). No differences were found regarding levels of physical activity or the number of conditions associated (*p* > 0.05) in any of the two groups described in the Table 3.

No notable associations were found at the technology use level, with both stable vs. unstable and regular vs. non-regular participants showing similar profiles (see Table 3). Intercountry differences regarding self-monitoring practices are described in Appendix A.2.

### 3.4. Unsupervised Analysis on Differentiating Factors of Self-Monitoring Practices

**Figure 4:**
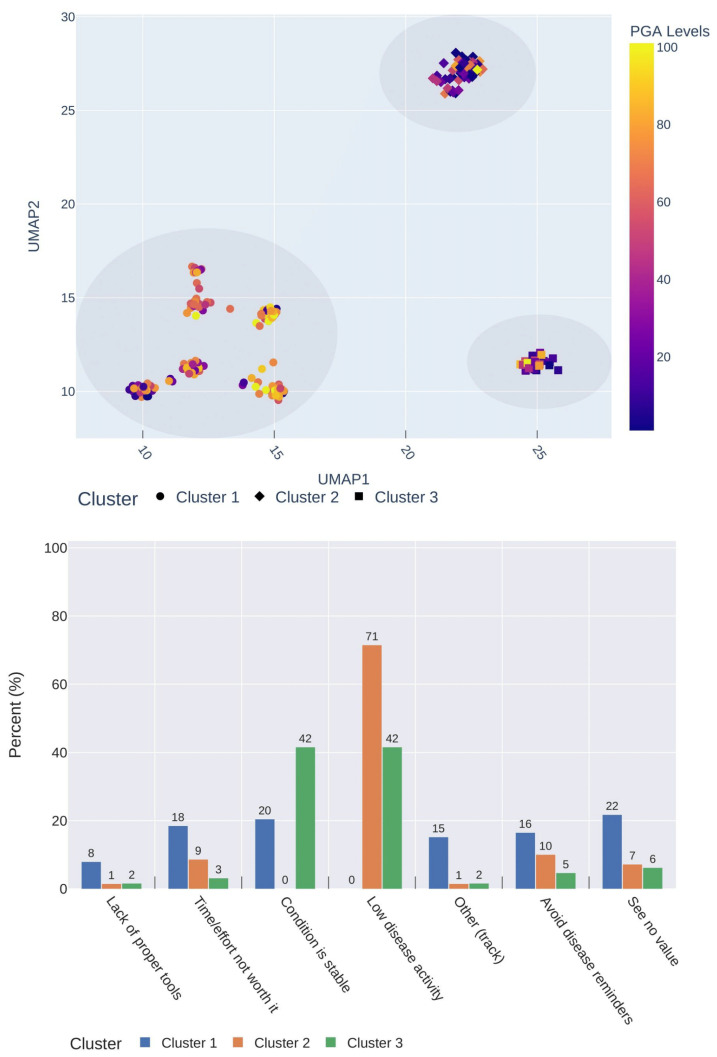
Two-dimensional projection of participants’ response describing self-monitoring profiles (**top**) and bar chart displaying the reasons explaining the lack of regular symptom monitoring for each cluster found (**bottom**). Dimensionality reduction was performed on the reasons that explained the lack of regularity in recording disease-related symptoms. The axes of the 2D-projection represent the UMAP components obtained after the dimensionality reduction process. The colour bar represents the PGA levels of each participant/sample (darker colour means better scores).

An unsupervised analysis was performed to assess the distinct response profiles to every item addressing self-monitoring practices of RMDs. Particularly, by analysing the reasons respondents described to explain the lack of regular symptom recording, three distinct clusters of responses were found (χ^2^ = 174.4; *p* < 0.001). After projecting such profiles into a 2D space, such clusters were clearly visible, as depicted by Figure 4 (top). Two of these clusters (2, 3) presented notably lower PGA levels (better health status), whereas the larger one (1) displayed generally worse levels.

In fact, by describing such response profiles of each cluster in a bar chart (Figure 4, bottom), most respondents belonging to cluster 2 (71%) and cluster 3 (84%) reported a stable condition and/or low disease activity as the main reasons explaining the absence of more regular tracking, which aligns with their better self-perceived health status. On the other hand, respondents from cluster 1 presented a sparser profile of responses than the remaining two, confirmed by the absence of prevalent reasons amongst them.

## 4. Discussion

This study investigated the self-monitoring practices of respondents diagnosed with RMDs, linking their habits with their self-perceived health status and technology adherence. Our findings suggest self-monitoring is generally accepted but still not consistently practiced, becoming more regular in multidisciplinary care scenarios and periods of disease decline. Even the willingness and interest to track symptoms is dependent on the perceived health status. Thus, this study complements prior work highlighting the benefits of using DHAs for self-monitoring [11,12,13,32] by presenting respondents’ actual monitoring measures and self-perceived benefits of using DHAs for monitoring disease progression, while observing sociodemographic, tech-related, and health-related behavioural patterns associated with barriers of usage reported in previous studies [10].

Participants’ agreement rates, either considering items about potential benefits of DHA or their most important features (see Figure 2) were high. Nevertheless, such rates are not reflected in the actual practices that were expected to be observed since 64% do not track symptoms regularly, 52% make use of non-digital means (e.g., mental notes), and 20% simply do not track. Participants appear willing to use these tools, but various factors hinder their adoption, including low disease activity (47%), perception of no additional value (15%), not being worth the effort (13%), and avoiding reminders about their condition (12%). For example, a survey study conducted during the COVID-19 pandemic [10] investigated the acceptance of DHAs between RMD patients and physicians. In this study, 90% of the patients agreed on using a digital health app for monitoring their condition, which is similar to our study (81.2%). In terms of the highlighted barriers of using apps, respondents reported there is too little information concerning suitable DHAs and the lack of usability. As in our study, participants appear to agree with the benefits of using DHA tools for self-monitoring their disease and improving clinical outcomes [11,32]. However, it is worth noting they did not use them particularly frequently (35–40% of regular usage) [32] due to low disease activity or no value perceived from monitoring. Thus, patient-centred design improvements and better engagement strategies (e.g., inviting patients to usability tests during early phases of DHA developments) may play an important role in raising the adherence levels from eagerness to effective use, leading to more stable usage solutions. Some projects are already including patients’ feedback in preliminary tests for mitigating such issues and to increase apps usability [20,25] but strong emphasis should be placed on the development of usable apps, meeting user needs and helping improve the care of patients with RMDs in the long-term.

Previous work already concluded that remote self-monitoring by patients improved self-care and disease outcomes [33], not only in RMD conditions [34] but also in other diseases, such as heart failure [35], diabetes [36], and hypertension [37]. By enabling a different perspective in understanding the disease progression and its mechanisms, it contributes to trustworthier clinical decision-making, which can ultimately help predict or detect disease flares or decline periods, while maintaining remission or at least low disease activity [18].

Our findings show consistent health-status profiles, emphasised by the fact that respondents who considered their disease was under control showed generally better QoL indicators and PGA levels, even when displaying a similar condition profile spectrum, which underlines the coherence between scales and self-perception of health status. Technology use was also identical between profiles but symptom recording practices were slightly distinct, with unstable participants recording symptoms more regularly. Respondents under self-reported stable condition cared less about disease monitoring. Such variations observed seem likely not to be caused by lack of technology adherence or digital literacy issues, but potentially by lack of engagement and awareness on the real benefits of using such apps.

This is in line with disease-related symptom recording practices. Participants who monitored at least monthly revealed generally worse health status scores (via EQ-5D, PGA levels) than those who barely recorded. A higher number of specialists involved in care correlated positively with regular symptom monitoring. This might again indicate that treatment is being carried out in a reactive rather than preventive fashion in individuals with high disease activity levels who adopt self-monitoring measures to keep track of their condition and improve their quality of life. Preventive treatment measures are lacking [38], as respondents’ adherence rates to track symptoms only increase upon a decline period. This might hold true since better perceived health status (low disease activity or condition under control) were the main reasons for not recording more regularly (see Figure 4, bottom). These respondents were more reluctant to use a mobile app for self-care purposes, producing an inversely proportional relationship between health status and disease self-monitoring. Those who recorded symptoms regularly used a wider range of devices to record symptoms, even though their general use of devices was similar to the participants not monitoring.

Demographic patterns also validated the consistency of the results. As participant’s age increases, the prevalence of multimorbidity [39] and mobility-related problems [40,41] is more likely to rise. In addition, although respondents from Austria described similar disease profiles as those from Portugal, in terms of number and type of RMDs, curiously, the former are embedded in a heterogenous care environment for disease management (more medical specialties). A strength of our study was that individuals living with RMDs were involved in the question items design, language adaptations, and dissemination. Nonetheless, associations found in this study would be more valid with a larger sample size. Moreover, as often seen in health surveys, the majority of participants had at least a high school degree level (90.3%), thus associations found in this study may not be applicable to a less literate population. Respondents were also not characterised by severe issues in terms of health status, which might have masked stronger associations that could arise in individuals with poorer QoL. This subject group might also be more health-conscious, as they were willing to participate in the survey (selection bias), which influences the generalisibility of the reports. Recall bias is another common factor potentially contributing to the presence of inaccurate self-reports; however, as most items captured routine-based patterns instead of sporadic events, we would expect a minor impact. Paper–pencil format was not considered for participation, which might have led to a more inclusive approach when recruiting.

## 5. Conclusions

Our study highlights the potential benefits of digital self-monitoring, with respondents agreeing on these in the most important aspects, while explaining their current practices. Self-monitoring practices were clearly more regular in participants being cared within a multidisciplinary team and with more compromised health status, where only half used digital means to monitor symptoms. While respondents expressed willingness to use digital tools, their adoption was hindered by factors such as (1) low disease activity (stable condition) and (2) no value perceived from tracking. To bridge this gap, patient-centred design improvements and better engagement strategies are crucial for transforming enthusiasm into effective, preventive use of these technologies in healthcare. As multidisciplinary teams foster stronger monitoring awareness, passive monitoring activities would provide healthcare providers with additional information independent of the individual’s view based on self-report items and may be deployed as a useful tool together with active monitoring.

## Figures and Tables

**Table 1:** Sociodemographic, condition, and quality of life characterisation of the survey participants (n (%) or M ± STD).

Dimension		Participant Group (n = 228)	
		n (%)/M STD	Missing Values, n (%)
Age	18–40	76 (33.3)	0 (0)
	40–60	115 (50.4)	
	>60	37 (16.2)	
Sex	Female	185 (81.1)	0 (0)
	Male	43 (18.9)	
	Diverse/Non-binary	0 (0.0)	
Country	Austria	114 (50.0)	0 (0)
	Germany	12 (5.3)	
	Ireland	11 (4.8)	
	Portugal	81 (35.5)	
	Other	10 (4.4)	
Education	No Formal	2 (0.9)	0 (0)
	Primary School	4 (1.8)	
	Lower Secondary	16 (7.0)	
	High School	88 (38.6)	
	Bachelor/Master	100 (43.8)	
	Doctorate	18 (7.9)	
Condition *	Osteoarthritis	23 (10.1)	0 (0)
	Osteoporosis	20 (8.8)	
	Psoriatic Arthritis	32 (14.0)	
	Spondylarthritis	41 (18.0)	
	Rheumatoid Arthritis	81 (35.5)	
	Systemic Sclerosis	10 (4.4)	
	Sjogren	25 (11.0)	
	Systemic Lupus	42 (18.4)	
	Vasculitis	11 (4.8)	
	Other	18 (7.9)	
Health-related QoL	EQ Mobility ^c^	1.9 ± 1.0	0 (0)
	EQ Self-Care ^c^	1.4 ± 0.8	
	EQ Usual Activities ^c^	2.1 ± 1.0	
	EQ Pain ^c^	2.4 ± 0.9	
	EQ Anxiety ^c^	1.9 ± 1.0	
	EQ-Index	0.8 ± 0.2	34 (14.5) ^a^
	EQ VAS ^d^	60.1 ± 27.3	15 (6.6) ^b^
	PGA ^e^	49.4 ± 31.2	0 (0)

*—multiple choice item (>100%); a—composite score computed using Portugal and Austria normative values; b—not answered by everyone; c—1 (no issues), 2 (slight issues), 3 (moderate issues), 4 (severe issues), 5 (extreme issues); d—scaled from 0 (worst) to 100 (best); e—scaled from 0 (best) to 100 (worst).

**Table 2:** Differences in self-monitoring practices of the participant group. Two groups of questions are addressed: (A) self-monitoring practices and (B) potential of digital technologies. Results are adjusted for age, sex, and education level. The reference group stands for “No”.

Variable		Disease under Control?				Monitor Symptoms Regularly?			
		Yes (n = 165)	No (n = 63)	*p*	OR/g	Yes (n = 82)	No (n = 146)	*p*	OR/g
A	How do you record? ^a^	1.2 ± 0.5	1.3 ± 0.6	>0.05	1.6 [0.9, 2.6]	1.4 ± 0.7	1.1 ± 0.4	**0.003**	2.4 [1.3, 4.3]
	When do you record? ^a^	1.6 ± 1.0	1.8 ± 1.3	>0.05	1.2 [0.9, 1.6]	2.4 ± 1.3	1.3 ± 0.7	**<0.001**	3.1 [2.2, 4.4]
	Why not record? ^a^	1.5 ± 0.8	1.3 ± 0.6	>0.05	0.7 [0.4, 1.1]	1.5 ± 0.8	1.4 ± 0.7	>0.05	1.3 [0.8, 1.9]
	Devices to record? ^a^	1.1 ± 0.4	1.1 ± 0.3	>0.05	0.4 [0.1, 1.2]	1.2 ± 0.4	1.1 ± 0.3	**0.004**	3.4 [1.5, 8.0]
	What useful for? ^a^	2.0 ± 0.9	2.1 ± 1.1	>0.05	1.1 [0.7, 1.9]	2.3 ± 1.0	1.8 ± 0.8	**0.015**	1.8 [1.1, 3.0]
	Recording frequency ^b^	4.8 ± 1.5	4.4 ± 1.6	**0.045**	0.8 [0.7, 1.0]	-	-	-	-
B	Use a monitoring app? ^c^	2.1 ± 1.7	1.8 ± 1.6	>0.05	0.9 [0.8, 1.1]	1.7 ± 1.3	2.3 ± 1.8	**0.008**	0.8 [0.6, 0.9]
	Share information with? ^a^	2.3 ± 1.7	2.7 ± 1.7	>0.05	1.1 [0.9, 1.3]	2.6 ± 1.6	2.3 ± 1.7	>0.05	1.1 [0.9, 1.3]

OR—Odds ratio (from logistic regression coefficients, β) with CI [2.5, 97.5] %/g—Hedges g effect size; a—number of items selected; b—1 (daily), 2 (two or three times/week), 3 (once a week), 4 (once a month), 5 (once in six months), 6 (never); c—1 (yes, definitely), 2 (yes, only advised by doctor), 3 (yes, only advised by family/friend), 4 (yes, only advised by doctor and family/friend), 5 (maybe, unsure), 6 (no).

**Table 3:** Differences on (A) the health status indicators and (B) the general technology use of the participant group. In EQ-5D-5L items, a higher score means more problems in that dimension and vice versa. In (B), a higher score means less usage (except the number of devices). Results are adjusted for age, sex, and education level. The reference group stands for “No”.

Variable		Disease under Control?				Monitor Symptoms Regularly?			
		Yes (n = 165)	No (n = 63)	*p*	OR/g	Yes (n = 82)	No (n = 146)	*p*	OR/g
A	EQ Mobility ^a^	1.7 ± 1.0	2.5 ± 1.0	**<0.001**	2.3 [1.6, 3.2]	2.1 ± 1.1	1.8 ± 0.9	**0.026**	1.4 [1.1, 1.9]
	EQ Self-Care ^a^	1.3 ± 0.6	1.9 ± 1.0	**<0.001**	2.9 [1.9, 4.4]	1.6 ± 0.9	1.3 ± 0.7	**0.033**	1.5 [1.1, 2.2]
	EQ Usual Activities ^a^	1.8 ± 0.9	2.7 ± 1.0	**<0.001**	2.7 [1.9, 3.8]	2.4 ± 1.1	1.9 ± 0.9	**0.003**	1.7 [1.3, 2.2]
	EQ Pain ^a^	2.1 ± 0.9	3.1 ± 0.7	**<0.001**	3.8 [2.5, 5.8]	2.6 ± 1.0	2.3 ± 2.0	>0.05	1.4 [1.0, 1.8]
	EQ Anxiety ^a^	1.8 ± 1.0	2.2 ± 1.1	**0.019**	1.4 [1.1, 1.9]	2.0 ± 1.1	1.8 ± 1.0	>0.05	1.2 [0.9, 1.6]
	EQ Index ^b^	0.9 ± 0.2	0.7 ± 0.3	**<0.001**	−0.7	0.8 ± 0.3	0.9 ± 0.2	**0.015**	0.4
	EQ VAS ^c^	68.0 ± 24.4	38.8 ± 23.3	**<0.001**	−1.2	53.7 ± 26.4	63.6 ± 27.3	**0.015**	0.4
	PGA ^c^	40.4 ± 30.1	72.8 ± 20.0	**<0.001**	1.1	55.0 ± 29.3	46.3 ± 31.9	**0.028**	−0.3
	Physical Activity ^d^	2.2 ± 0.8	1.8 ± 0.8	**0.026**	0.6 [0.4, 1.0]	1.9 ± 0.8	2.1 ± 0.8	>0.05	0.7 [0.5, 1.0]
	No. conditions	1.4 ± 0.9	1.6 ± 0.9	>0.05	1.2 [0.9, 1.6]	1.4 ± 0.7	1.5 ± 1.0	>0.05	0.8 [0.5, 1.1]
	No. specialists	2.7 ± 1.6	2.9 ± 1.7	>0.05	1.0 [0.9, 1.2]	3.3 ± 1.7	2.5 ± 1.5	**<0.001**	1.4 [1.1, 1.6]
B	Internet Access ^e^	1.1 ± 0.5	1.2 ± 0.7	>0.05	1.2 [0.7, 2.1]	1.2 ± 0.7	1.1 ± 0.5	>0.05	1.5 [0.9, 2.4]
	Smartphone Usage ^e^	1.1 ± 0.6	1.2 ± 0.8	>0.05	1.2 [0.8, 2.0]	1.2 ± 0.8	1.1 ± 0.5	>0.05	1.3 [0.8, 2.0]
	Tablet Usage ^e^	3.5 ± 1.7	3.7 ± 1.6	>0.05	1.0 [0.8, 1.2]	3.2 ± 1.7	3.8 ± 1.6	**0.050**	0.8 [0.7, 1.0]
	Computer Usage ^e^	1.8 ± 1.3	2.2 ± 1.6	>0.05	1.1 [0.9, 1.4]	1.7 ± 1.3	2.1 ± 1.4	>0.05	0.8 [0.7, 1.1]
	No. devices	2.1 ± 0.7	1.9 ± 0.7	>0.05	0.8 [0.5, 1.2]	2.3 ± 0.8	2.0 ± 0.7	0.050	1.4 [1.0, 2.2]

OR—Odds ratio (from logistic regression coefficients, β) with CI [2.5, 97.5]%/g—Hedges g effect size. a—1 (no issues), 2 (slight issues), 3 (moderate issues), 4 (severe issues), 5 (extreme issues); b—N = 195 (Portuguese and Austrian); c—Scaled from 0 to 100 (EQ VAS and PGA); d—1 (low, <30 min exercise/week), 2 (moderate, >30 min exercise/week), 3 (high, >1 h intense exercise/week); e—1 (multiple times/day), 2 (few times/week), 3 (weekly), 4 (monthly), 5 (no use at all).

**Figure A1:**
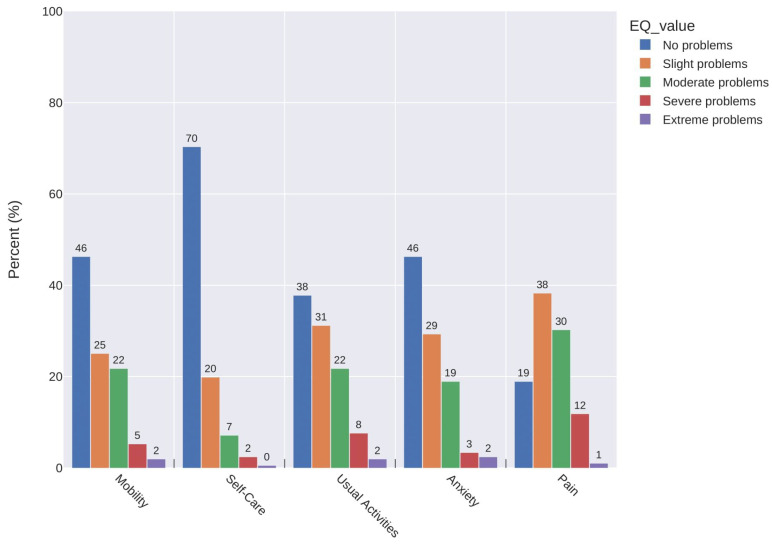
EQ-5D-5L dimension scores displayed in terms of the problem severity reported by the participant group (from “no” to “extreme” problems).

## Data Availability

Data will be made available upon request to the corresponding author.

## Appendix A

### Appendix A.1. Health-Related Quality of Life

Most participants reported no to moderate difficulties in most of the EQ-5D-5L dimensions (see Figure A1). In terms of mobility, around 71% of participants reported no or slight difficulties walking. Most respondents also revealed no or slight problems regarding self-care, usual activities, and anxiety dimensions (>69%). Worse results were described in the dimension of pain, as 42% of participants reported moderate to extreme pain/discomfort. Scores of the EQ-VAS scale displayed a broad range (mean: 60.1 ± 27.3, where higher scores mean better health status), alongside the PGA assessment scores (mean: 49.4 ± 31.2, where lower scores indicate lower disease activity), with both pointing more towards a good health status condition. These two measures were found to be strongly (but inversely) correlated with each other (*p* < 0.001, r = −0.66), assuring construct assertiveness (see Figure A1). Physical activity levels were varied: 29% of the participants reported low activity (<30 min/week), 37% described moderate activity (>30 min/week), and 34% showed intense activity levels (>1 h/week).

### Appendix A.2. Self-Monitoring Practices Concerning Country Differences

There were some differences found between participants from Austria and Portugal, the most common countries of respondents (n = 195). These two countries slightly differed in terms of age (*p* = 0.010) and sex (*p* = 0.019), as the Austrian cohort (n = 114, 90.1% women, 43.8% < 40 years-old) was slightly younger and incorporated more female participants than the Portuguese (n = 81, 77.2% women, 18.5% < 40 years-old). In terms of self-monitoring practices, Austrian respondents were found to be willing to share their app-recorded symptoms with more people (including healthcare professionals) than Portuguese (OR: 1.5 [1.2, 1.9], *p* < 0.001), possibly due to a higher number of specialists involved in their care (OR: 3.2 [2.2, 4.5], *p* < 0.001). Austrian participants also used smartphones more often to track health and condition-related symptoms (OR: 2.6 [1.1, 5.9], *p* = 0.028). This did not translate into better self-monitoring practices, as the frequency of recording symptoms and the adherence rate to a condition-supporting app did not vary between these two countries.

As expected, the health status also varied geographically. In fact, Austrian participants reported more intense activity levels than the Portuguese sub-group (OR: 4.2 [2.6, 6.8], *p* < 0.001). Moreover, the former experienced fewer issues with pain (OR: 0.6 [0.5, 0.9], *p* = 0.006) and lower PGA levels (g = −0.4, *p* = 0.013), in addition to a better health status (g = 0.4, *p* = 0.026). Compared to normative values, EQ-index scores also revealed significant differences, with Austrian participants generally scoring lower than Portuguese (g = −0.5, *p* = 0.017). Although with slightly distinct age profiles, these countries did not reveal any differences in the number of diagnosed conditions (*p* > 0.05).

The use of technology for general purposes revealed that Austrians selected more devices (OR: 1.6 [1.1, 2.6], *p* = 0.030), particularly, more tablet usage (OR: 0.8 [0.6, 0.9], *p* = 0.008).
